# The Sage Resource Project: Readying Researchers to Transform Research through Engagement

**DOI:** 10.1093/geroni/igab046.575

**Published:** 2021-12-17

**Authors:** Rachel Lessem, Rebecca Berman, Jesse Bella, Margaret Danilovich

**Affiliations:** 1 CJE SeniorLife, Chicago, Illinois, United States; 2 Leonard Schanfield Research Institute, CJE SeniorLife, Illinois, United States; 3 Gerontology Department, Northeastern Illinois University, Illinois, United States; 4 CJE SeniorLife, Skokie, Illinois, United States

## Abstract

The Sage Model enables engagement of older adults receiving Long Term Services and Supports (LTSS), a group typically excluded in research. This presentation focuses on lessons learned from The Sage Resource Project, a Patient Centered Outcomes Research Institute funded project. We collaborated with RCMAR and Roybal centers to encourage NIH-affiliated researchers to embrace stakeholder engagement through promotion of the Sage Model. Few studies include an assessment of researcher needs when it comes to stakeholder engagement. We conducted a needs assessment (n=103) finding <50% of researchers presented work to older adults and only 41% interacted with older adults receiving LTSS. However, >90% were likely to attend webinars to learn more. Additionally, 70% of respondents were interested in setting up their own Sage Model research advisory boards. We identify opportunities for transforming LTSS research by including older adults as well as directions for future research on engagement, based on researchers’ identified needs.

